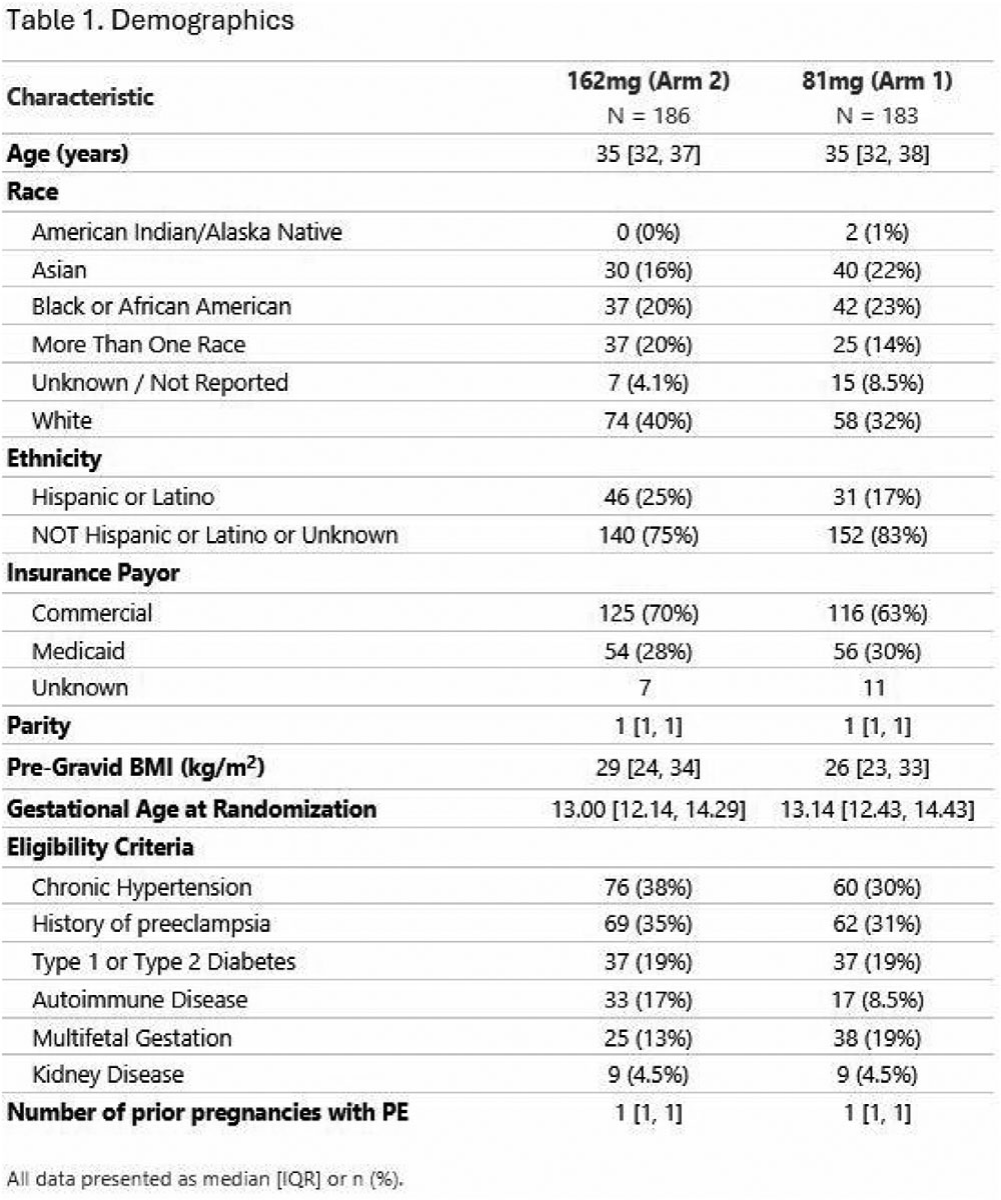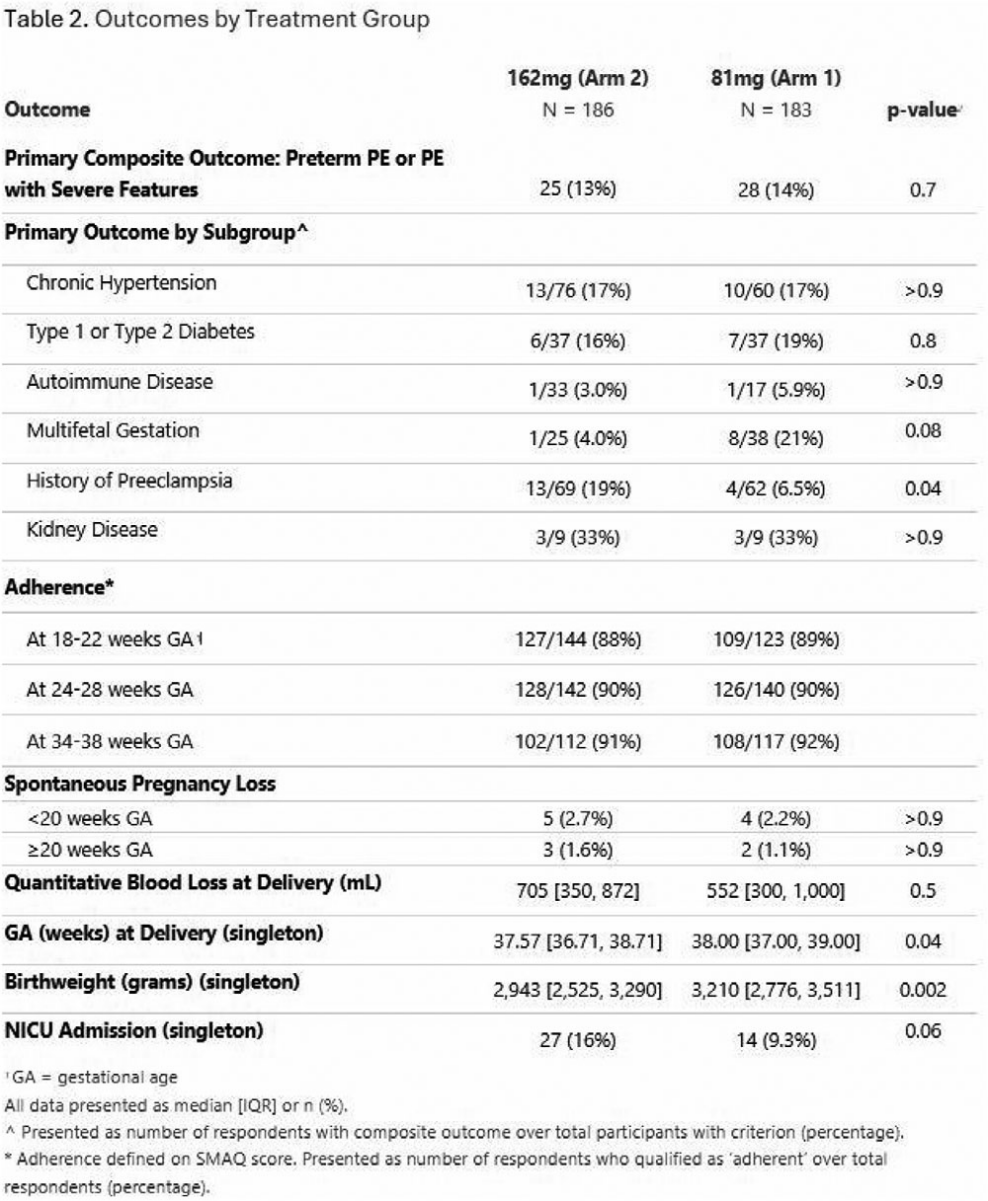# Late‐Breaking Abstract Presentations

**DOI:** 10.1002/pmf2.12035

**Published:** 2025-01-05

## LB01 CErebro Placental RAtio based Management in Reduced Fetal Movements; an International Cluster‐Randomized Clinical Trial (CEPRA)

Laura A. Lens^1^; Selina Posthuma^2^; Stefanie E. Damhuis^3^; Renée J. Burger, N/A^1^; Henk Groen^4^; Ruben G. Duijnhoven^5^; Sailesh Kumar^6^; Alexander E.P Heazell^7^; Asma Khalil^8^; Wessel Ganzevoort^9^; Sanne J. Gordijn^10^

*^1^Amsterdam UMC, Amsterdam, Noord‐Holland; ^2^Uiversity Medical Center Groningen, University Medical Center Groningen, Groningen; ^3^Amsterdam University Medical Centers, Amsterdam University Medical Centers, Noord‐Holland; ^4^University Medical Center of Groningen, University Medical Center of Groningen, Groningen; ^5^Amsterdam UMC, location University of Amsterdam, Amsterdam, Noord‐Holland; ^6^The University of Queensland, Brisbane, Queensland; ^7^The University of Manchester, Manchester, England; ^8^Fetal Medicine Unit, St George's Hospital, St George's University of London, Fetal Medicine Unit, St George's Hospital, St George's University of London, England; ^9^Amsterdam University Medical Centers, Amsterdam, Groningen; ^10^University Medical Center Groningen, Groningen, Groningen*

10:00 AM ‐ 10:15 AM

**Objective**: Routine assessments in near‐term pregnancies often fail to accurately detect fetal compromise due to placental insufficiency, especially in non‐small for gestational age fetuses. The cerebroplacental ratio (CPR) serves as a potential indicator of placental insufficiency and adverse outcomes. This study aims to evaluate whether expedited birth in women with reduced fetal movements, potentially a sign of placental insufficiency, and low CPR improves neonatal outcomes.

**Study Design**: In this international, multicenter, cluster‐randomized controlled trial, we randomly assigned 22 Dutch and 1 Australian hospitals to CPR‐based management (i.e. expedited birth in case of CPR < 1.1) and concealed CPR measurement with routine clinical care in case of reduced fetal movements at term (37+0 to 40+6 weeks’ gestation). Women were eligible to participate in the trial if the fetus was estimated to be above the 10^th^ percentile, if cardiotocography was normal, and if there were no other reasons necessitating expedited birth within 4 days. Primary outcome was a composite of severe adverse perinatal outcomes: stillbirth, neonatal mortality, 5‐minute Apgar score < 7, umbilical artery pH < 7.10, emergency birth for fetal distress and/or severe neonatal morbidity.

**Results**: From July 2020 to September 2024, 1816 women participated in the trial. 1676 Women with complete data were included in the intention to treat analysis. In preliminary data, the composite of severe adverse perinatal outcomes occurred in 99 (11.7%) of 849 participants who received CPR‐based management versus in 127 (15.4%) of 827 participants in the concealed CPR group (relative risk 0.76; 95% confidence interval 0.58 to 0.99, Figure 1, Table 1). This was mainly driven by a reduction in severe neonatal morbidity, Apgar score < 7, umbilical artery pH < 7.10 and/or emergency birth for fetal distress.

**Conclusion**: In women presenting with perceived reduced fetal movements at term in non‐small for gestational age fetuses, CPR‐based management, i.e. expedited delivery if CPR < 1.1 and expectant management if CPR > 1.1, reduced severe adverse perinatal outcomes.

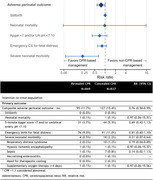

## LB02 iSEARCH RCT: Evaluating the Effectiveness of Maternal Sildenafil to Reduce Complications Related to Intrapartum Hypoxia

Sailesh Kumar^1^; Ben W. Mol^2^; William Tarnow‐Mordi^3^; Jon Hyett^4^; Nadia Badawi^4^; Annalene Seidler^5^; Helen Liley^6^; Emily Callander^7^; Vicky Flenady^6^; Sue Walker^8^; Rachel O'Connell^4^

*^1^The University of Queensland, Brisbane, Queensland; ^2^Monash University, Clayton, Victoria; ^3^The University of Sydney, Sydney, New South Wales; ^4^University of Sydney, Sydney, New South Wales; ^5^Medical Uni Rostock, Rostock, Mecklenburg‐Vorpommern; ^6^Mater Research Institute‐University of Queensland, Brisbane, Queensland; ^7^University Technology Sydney, Sydney, New South Wales; ^8^Department of Obstetrics, Gynaecology and Newborn Health, University of Melbourne, Melbourne, Victoria*

10:15 AM ‐ 10:30 AM

**Objective**: Sildenafil citrate (SC) has been proposed as a drug to reduce fetal distress during labour. We assessed its effectiveness in a large placebo controlled clinical trial.

**Study Design**: This was a double‐blind multicentre randomised controlled trial (Trial Registration Number ACTRN12621000231842) funded by the Australian Medical Research Future Fund. We randomised women attempting vaginal birth at term to 50mg SC every 8 hours during labour (maximum of 3 doses) or placebo. Primary outcome was a composite of 10 adverse neonatal outcomes (intrapartum stillbirth, neonatal death, Apgar score < 4 at 5 minutes, cord artery pH < 7.0, neonatal encephalopathy, neonatal seizures, neonatal respiratory support >4 hours, neonatal unit admission > 48hours, persistent pulmonary hypertension of the newborn and meconium aspiration syndrome). Secondary outcomes were caesarean section (CS) or instrumental vaginal birth (IVB) for fetal distress. The sample size of 3200 women was predicated on a 35% reduction in the RR of the composite perinatal outcome by 35% (7% to 4.55%).

**Results**: Between 7 September 2021 and 30 June 2024 we randomized 3257 women (including 18 sets of twins) to SC (1626) and placebo (1631). Induction of labour occurred in 83.5% of the SC cohort and 82.9% of the placebo group. Electronic fetal heart rate monitoring rates were similar (97.4%) in both arms. The primary outcome occurred in 5.1% of women taking SC and 5.2% of those taking placebo [RR 1.02 (0.75‐1.37)] with no differences in any of the components of the composite outcome. There was also no difference in rates of CS or IVB for fetal distress  [21.1% vs. 18.8%, RR 1.12 (0.98‐1.29)]. Blood loss >1000ml occurred in 10.1% in the SC group vs. 7.9% [RR1.29, (1.03‐1.60)].

**Conclusion**: Maternal oral SC in women undergoing term labour did not result in a reduction of adverse neonatal outcomes potentially related to intrapartum hypoxia.

## LB03 Single‐Dose Intravenous Iron Versus Oral Iron for Maternal Iron Deficiency Anemia: A Randomized Controlled Trial

Richard Derman^1^; Mrutyunjaya Bellad^2^; Manjunath Somannavar^2^; Sudhir Bhandari^3^; Sudhir Mehta^3^; Seema Mehta^3^; Dharmesh Sharma^3^; Yogesh Kumar^4^; Umesh Charantimath^4^; Amaresh Patil^5^; Ashalata Mallapur^6^; Umesh Ramdurg^6^; Radha Sangavi^7^; Praveen Patil^7^; Subarana Roy^8^; Phaniraj Vastrad^9^; Chander Shekhar^10^; Benjamin Leiby^10^; Rebecca Hartman^10^; Michael Georgieff^11^; Stephen Mennemeyer^10^; Zubair H. Aghai^12^; Simal Thind^10^; Rupsa C. Boelig^13^

*^1^Jefferson Health, Philadelphia, PA; ^2^KLE Academy of Higher Education and Research's J N Medical College,, Belagavi, Karnataka; ^3^Sawai Man Singh Medical College, Jaipur, Rajasthan; ^4^KLE Academy of Higher Education and Research's J N Medical College,, KLE Academy of Higher Education and Research's J N Medical College,, Karnataka; ^5^KLE Academy of Higher Education and Research's J N Medical College, Belagavi, Karnataka; ^6^S Nijalingappa Medical and HSK Hospital and Research Center, Bagalkote, Karnataka; ^7^Raichur Institute of Medical Sciences, Raichur, Karnataka; ^8^ICMR – National Institute of Traditional Medicine, Belagavi, Karnataka; ^9^Model Rural Health Research Unit, Sirwar, Karnataka; ^10^Thomas Jefferson University, Philadelphia, PA; ^11^University of Minnesota, Minneapolis, MN; ^12^Sidney Kimmel Medical College at Thomas Jefferson University, Philadelphia, PA; ^13^Sidney Kimmel Medical College, Thomas Jefferson University, Philadelphia, PA*

10:00 AM ‐ 10:15 AM

**Objective**: Maternal iron deficiency anaemia (IDA) is a persistent global health challenge with increased risk of adverse perinatal outcomes. Obstetric guidelines recommend first line treatment with oral iron, however rates of maternal IDA remain above global targets. We aimed to determine whether single dose intravenous (IV) iron for maternal IDA in the 2nd trimester was superior to oral iron in reducing incidence of low birth weight (LBW) infants and maternal anemia at delivery.

**Study Design**: This is a parallel, three‐arm superiority randomized controlled multi‐center trial in India of pregnant singletons at 14‐17 weeks with moderate IDA randomized to: (1) 60mg oral ferrous sulphate twice daily; or single dose infusion of (2) intravenous (IV) ferric derisomaltose (FDM) or (3) IV ferric carboxymaltose (FCM). The dual primary outcomes were: (1) LBW (< 2500 grams [g]) and (2) maternal non‐anemic state (NAS) (Hb >11·0 g/dL at 30‐34 weeks or delivery). Participants received rescue treatment per local clinical care guidelines (Hb< 7g/dL or < 1g/dL improvement in Hb). Sensitivity analysis performed defining NAS as Hb≥11.0 without additional non‐study iron or blood transfusion.

**Results**: The oral iron, FDM, and FCM arms included 1450, 1456, and 1462 participants respectively. There was a reduced rate of LBW with IV FCM (25·2%, Relative Risk [RR] 0·87 [97·55% CI 0·75,0·99]), but not IV FDM (29·1%, RR 0·98 [97·55% CI 0·86,1·12]) vs oral iron (Fig1). Achievement of NAS was not improved: IV FCM (RR 1·05 [99·95% CI 0·97‐1·15]) and IV FDM (RR 1·06 [99·95% CI 0·98, 1·16]) vs oral (Fig 1, 2A). In sensitivity analysis, there was increased rate of NAS in both IV FDM (RR 1.25 (1.13‐1.396), p< 0.0001) and IV FCM (RR 1.24 (1.12‐1.38), p< 0.0001) vs oral (Fig 2C).

**Conclusion**: First‐line treatment of maternal IDA with single dose infusion of IV iron results in a reduced incidence of LBW infants (IV FCM) and higher incidence of maternal NAS without additional iron or blood transfusion (IV FCM and FDM).

Clinical guidelines should address the potential benefit of single dose IV iron as the primary treatment of IDA in pregnancy.

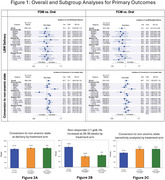

## LB04 Neonatal Impact of Prematurity Risk Biomarker Screening with Targeted Interventions: A Multicenter Randomized Controlled Trial

Brian K. Iriye^1^; John O'Brien^2^; Christopher S. Ennen^3^; Perry Barrilleaux^4^; Jill Berkin^5^; Anna Palatnik^6^; Moeun Son^7^; Cynthia Gyamfi‐Bannerman^8^; Mollie McDonald^9^; Glenn Markenson^10^; Anthony C. Sciscione^11^; Joseph R. Biggio, Jr^12^; Samuel Wolf^13^; Scott Sullivan^14^; Michael Walker^15^; Babak Shahbaba^16^; Stephen P. Pound^17^

*^1^Hera Womens Health, Las Vegas, NV; ^2^University of Kentucky, Lexington, KY; ^3^University of Virginia School of Medicine, Charlottesville, VA; ^4^Ochsner Louisiana State University Health Shreveport, Shreveport, LA; ^5^Icahn School of Medicine at Mount Sinai, New York, NY; ^6^Medical College of Wisconsin, Milwaukee, WI; ^7^Weill Cornell Medicine, New York, NY; ^8^University of California, San Diego, San Diego, CA; ^9^Austin Maternal‐Fetal Medicine, Austin, TX; ^10^Boston Medical Center, Boston, MA; ^11^Christiana Care Health System, Newark, DE; ^12^Ochsner Health, New Orleans, LA; ^13^Emerald Coast OBGYN Clinical Research, Panama City, FL; ^14^Inova Fairfax Medical Campus, Fairfax, VA; ^15^Walker Bioscience, Carlsbad, CA; ^16^University of California, Irvine, Irvine, CA; ^17^Virginia Physicians for Women, North Chesterfield, VA*

10:15 AM ‐ 10:30 AM

**Objective**: Obstetric advances have had limited impact on preterm birth (PTB) rates and neonatal outcomes. We assessed outcomes in a cohort of average risk singleton pregnancies using biomarker‐based PTB risk stratification, followed by interventions for those identified at higher risk. 

**Study Design**: This randomized controlled trial enrolled 5,018 singleton pregnancies at 19 U.S. centers from 2020‐2023. Individuals with prior PTB, short cervix, severe maternal conditions, or known genetic/fetal anomalies were excluded. We measured the ratio of maternal circulating insulin‐like growth factor‐binding protein 4 to sex hormone‐binding globulin between 18w0d and 20w6d, with participants randomized 1:1. The control arm, blinded to test results, received usual care, while the prevention arm received test results. Prevention arm participants identified as high‐risk were offered a regimen of daily vaginal progesterone, low‐dose aspirin, and weekly nursing calls. Lower‐risk participants in the prevention arm received usual care. Follow‐up continued through delivery and neonatal discharge. The prespecified modified intent‐to‐treat (mITT) analysis included the control group, prevention arm low‐risk participants, and high‐risk participants who consented to intervention by 24 weeks, while excluding those hospitalized with COVID‐19. Primary outcomes included neonatal length of stay (NNLOS) and a composite morbidity index (NMI). A planned intent‐to‐treat (ITT) analysis assessed multiple neonatal outcomes.

**Results**: Baseline characteristics did not differ between arms. mITT analysis of co‐primary outcomes (Table 1) showed significantly decreased NMI and NNLOS in the prevention arm. The ITT analysis displayed significant reductions in NMI (20%), NNLOS (8%), neonatal intensive care unit (NICU) length of stay (6%), and NICU admission (20%) (Table 2). 

**Conclusion**: In an average risk singleton population, a mid‐trimester blood test identified pregnancies at higher PTB risk, and implementing the treatment bundle significantly reduced neonatal morbidity and hospital stay compared to usual care.

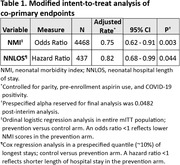

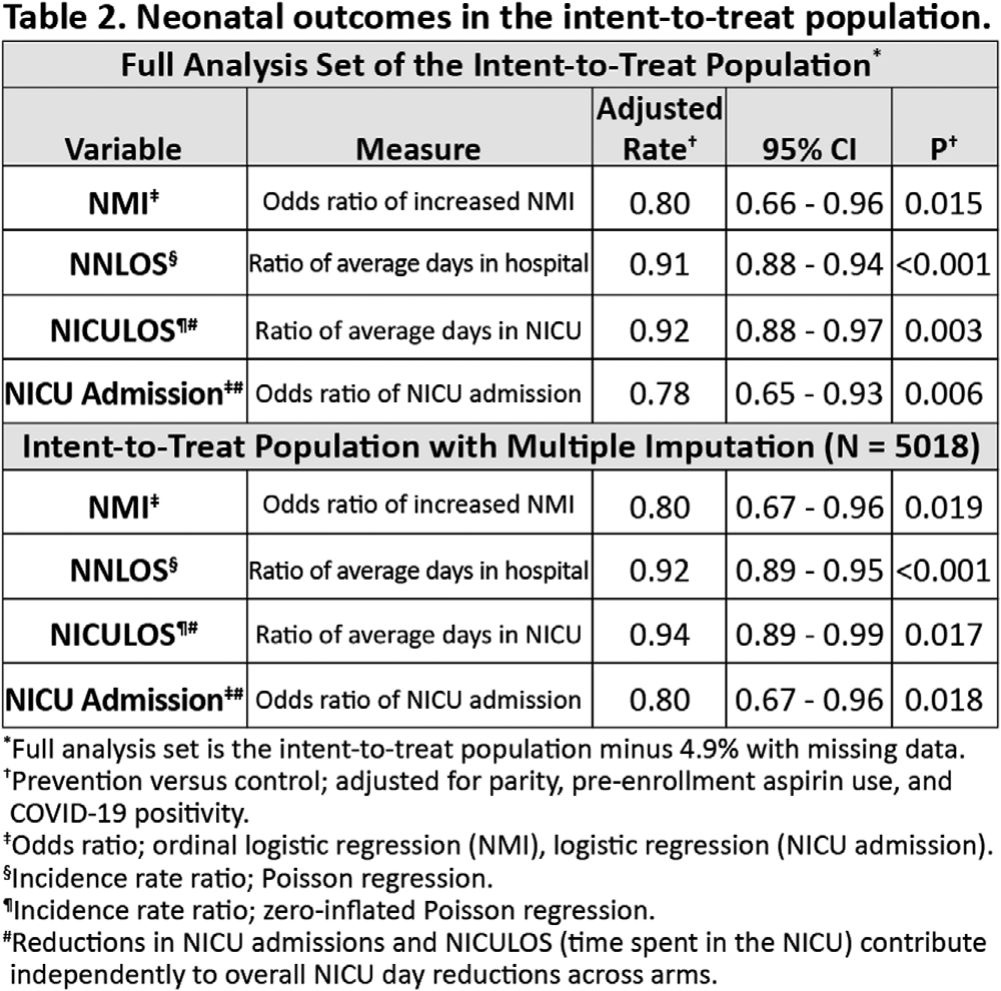

## LB05 Patient Navigation to Improve Postpartum Health Care Among Low‐Income Birthing People: A Randomized Controlled Trial

Lynn M. Yee^1^; Joe M. Feinglass^1^; Brittney R. Williams^1^; Laura Diaz^1^; Viridiana Carmona‐Barrera^1^; Ying Cheung^1^; Ka'Derricka Davis^1^; Chen Yeh^1^; Charlotte M. Niznik^1^; Michelle A. Kominiarek^2^; William A. Grobman^3^

*^1^Northwestern University Feinberg School of Medicine, Chicago, IL; ^2^Northwestern Feinberg School of Medicine, Northwestern Feinberg School of Medicine/ Chicago, IL; ^3^Women & Infants Hospital of Rhode Island / Alpert Medical School of Brown University, Brown University/Providence, RI*

10:15 AM ‐ 10:30 AM

**Objective**: To evaluate whether postpartum (PP) patient navigation (PN) improves PP health care quality among birthing people with low income.

**Study Design**: In this single‐center RCT, English‐ or Spanish‐speaking pregnant people aged 16 or older with Medicaid were randomly assigned between 30 weeks of gestation and PP discharge to one year of PN versus usual care. Participants assigned to PN were partnered with a trained lay navigator who provided individualized, intensive PN services designed around PP‐specific barrier ascertainment and reduction. PN services included social needs assessments and support, healthcare appointment and insurance assistance, health education, and support for engagement in shared decision making. Data were collected via patient report and medical record abstraction at enrollment, 4‐12 weeks PP, and 11‐13 months PP. The primary outcome was achievement of a six‐component composite measure representing receipt of optimal PP care by 12 weeks PP (Table 1). Secondary outcomes included a PP care score (% of components achieved), each component of optimal PP care, and 11‐13 month health services outcomes.

**Results**: From January 2020 to June 2023, 405 people were randomized (203 PN, 202 usual care) of whom 50% identified as non‐Hispanic Black and 41% as Hispanic. The primary outcome of optimal PP care was similar between groups (9.4% PN vs. 7.9% usual care, p = 0.6); however, the mean PP care score was significantly higher among PN recipients (72% ±17% vs. 64% ±23%, p = 0.001). Three components drove this difference: PP visit completion (96% vs. 80%, p< 0.001), receipt of all indicated anticipatory guidance (65% vs. 51%, p = 0.008), and PP depression screening/care (86% vs. 72%, p< 0.001). At 11‐13 months, PN recipients were significantly more likely to be using their desired family planning method and have attended a primary care appointment (Table 2).

**Conclusion**: The primary outcome, a composite measure of optimal PP care, was not different between groups but was achieved infrequently. However, PN improved several aspects of PP care quality and increases frequency of transition to primary care.

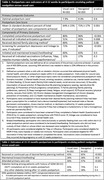

## LB06 Comparing 162mg vs. 81mg Aspirin for Prevention of Preeclampsia (ASAPP): A Randomized Control Trial

Amrin Khander^1^; Kathy Matthews^2^; Charlene Thomas^3^; Paul Christos^4^; Stephen T. Chasen^5^; Daniel W. Skupski^3^; Laura E. Riley^3^; Phyllis August^6^; Line Malha^3^

*^1^Weill Cornell Medicine‐New York Presbyterian Hospital, New York, NY, NY; ^2^New Jersey Perinatal Associates, Livingston, NJ; ^3^Weill Cornell Medicine, New York, NY; ^4^Weill Cornell Medical College, New York, NY; ^5^Weill‐Cornell Medical College, New York, NY; ^6^New York Presbyterian Weill Cornell Medicine, New York, NY*

10:15 AM ‐ 10:30 AM

**Objective**: Low dose aspirin (ASA) < 100 mg vs placebo reduces the incidence of preterm preeclampsia (PE) by 20‐30% for those at high risk. Doses 100‐150mg may further reduce the incidence by 60% compared to placebo.  We compared 162mg to 81mg of ASA per day in the prevention of PE in high‐risk pregnancies.

**Study Design**: We conducted a pragmatic prospective randomized open label blinded‐endpoint (PROBE) clinical trial. Pregnant persons at high risk for PE (as defined by ACOG) with one or more of the following criteria: chronic hypertension, history of PE, diabetes mellitus, autoimmune disease, multifetal gestation or kidney disease were randomized (< 16weeks) to either 81mg or 162mg ASA daily and monitored throughout pregnancy (20±2 weeks, 26±2 weeks, and 36±2 weeks) until 6 weeks postpartum. The primary outcome was a composite of developing preterm PE (< 37 weeks) or PE with severe features. Outcomes were adjudicated by independent investigators blinded to treatment group and clinical diagnosis. Secondary outcomes were adherence to therapy (determined by a validated Simplified Medication Adherence Questionnaire), maternal and fetal complications. Based on a prespecified interim analysis of the first 100 participants, we estimated that 400 participants would be required to detect a 7.1% difference in the primary outcome between the groups.

**Results**: Of 400 participants randomized, 369 had delivery data and were included in the intention to treat analysis (17 lost to follow up/withdrew, 14 elective terminations) with 186 participants in the 162mg and 183 in the 81mg group. Baseline characteristics were similar. The incidence of preterm PE or PE with severe features was 25/183 (13%) in the 162mg group vs. 28/180 (14%) in the 81mg group (p = 0.7). Adherence rates ranged from 88%‐91% vs 89%‐92% for the 162mg vs 81mg groups respectively across study visits. Infant birthweight was slightly lower in the 162 mg group (2.9 kg vs 3.2 kg; p = 0.002).

**Conclusion**: Our data show that the rates of preterm PE or PE with severe features were similar in pregnant people at high risk for PE taking either 81mg or 162mg ASA.